# Factors associated with delayed first ophthalmological consultation for primary glaucoma: a qualitative interview study

**DOI:** 10.3389/fmed.2023.1161980

**Published:** 2023-07-17

**Authors:** Hua Liu, Chen Chen, Zhuo Chen, Qian Li, Quan Li, Wei Liu

**Affiliations:** Tianjin Key Laboratory of Retinal Functions and Diseases, Tianjin Branch of National Clinical Research Center for Ocular Disease, Eye Institute and School of Optometry, Tianjin Medical University Eye Hospital, Tianjin, China

**Keywords:** delayed consultation, glaucoma, medical care behavior, occult symptoms, visual field damage

## Abstract

**Background:**

Glaucoma has an insidious onset with non-specific early symptoms, often leading patients to delay in seeking help. However, postponing the first ophthalmological consultation can result in delayed diagnosis and treatment, with adverse effects on vision. This study explored the factors associated with delayed first ophthalmological consultation in patients with primary glaucoma, with the overarching aim of informing measures to reduce delayed consultation and avoid the consequent adverse outcomes.

**Methods:**

We adopted a phenomenological approach. Semi-structured interviews were conducted with patients admitted to a tertiary eye hospital in Tianjin, China, from January 2021 to April 2021. Data were analyzed by Colaizzi’s seven-step method.

**Results:**

We identified 46 patients with primary glaucoma who delayed their first ophthalmological consultation for various reasons. There were four major themes and 16 sub-themes. The major themes were as follows: (1) occult symptoms that are difficult to identify; (2) insufficient knowledge and understanding of glaucoma-related risks and harm; (3) perceived difficulties in accessing medical care; and (4) inadequate support system.

**Conclusion:**

In order to avoid patient delay and consequent irreversible damage to the visual field in patients with primary glaucoma, it is essential that medical staff identify symptoms more effectively, change habitual medical behavior of the patients, adopt a medical union model, and promote the use of a social medical support system to address practical difficulties in delivering adequate care.

## Background

Glaucoma is the main cause of irreversible blindness globally, with pathological elevated intraocular pressure being the major risk factor. Elevated intraocular pressure levels and the intolerance of the optic nerve to this pressure lead to optic nerve atrophy and visual field defects (1). Glaucoma was estimated to have affected 79.6 million people worldwide in 2020, and this number is anticipated to increase to 111.8 million in 2040 (2, 3), with Asia proposed to have the highest incidence (4).

Notably, approximately 50% of patients are not aware that they have glaucoma (5). Glaucoma poses a serious threat to vision, as its development is insidious and the early symptoms are atypical. Clinical manifestations of glaucoma overlap with those of few degenerative brain and digestive system diseases (6). In order to improve the early diagnosis and treatment of glaucoma, efforts have been made to construct predictive genetic risk models (7) and a glaucoma self-reporting system (8), and establish the relationship between the risk assessment of systemic diseases and glaucoma (9). Nevertheless, these approaches have been unable to significantly improve delays in first ophthalmological consultation for primary glaucoma.

In 1946, Pack and Gallo first described the concept of “patient delay,” in which a patient delays seeking help. A delay of ≥3 months has been defined as an undue delay (10). Eissa et al. (11) divided the total delay into three stages: patient delay, diagnosis delay, and treatment delay. Prior et al. (12) divided the medical treatment delay into patient delay and healthcare provider delay. Patient delay refers to a delay in seeking care by the patient, while healthcare provider delay includes both detection delay and service delay. Detection delay refers to a delay in investigations by a healthcare professional to diagnose glaucoma. Service delay refers to a delay in referring the patient to a glaucoma specialist and a delay in glaucoma treatment.

A high proportion of patients with glaucoma experience irreversible damage to their vision due to a delay in their first ophthalmological consultation. Jones et al. (13) investigated 10,766 patients with glaucoma who attended a glaucoma clinic for the first time in Britain and Tanzania. Data from the British glaucoma clinic showed that 4.6% of patients already had severe visual field damage in one or both eyes. Data from Tanzania were even more alarming, with 44.7% of patients already having severe visual field damage in one or both eyes. Other epidemiological data from Africa showed that the proportion of glaucoma cases with delayed first ophthalmological consultation was as high as 50%; the degree of visual impairment in severe cases had already reached the level of blindness (14). Thus, as glaucoma is a chronic ophthalmic disease, early detection and treatment is crucial to retard progressive damage to the visual field, ensure patient quality of life, conserve medical resources, and reduce the burden of care imposed by the onset of blindness.

At present, factors associated with delays in first ophthalmological consultation in patients with glaucoma are unclear. Therefore, the objective of this study was to determine causes of first ophthalmological consultation delay by interviewing patients with glaucoma who had previously delayed their first ophthalmological consultation. Determination of the underlying factors for such delays would facilitate improvements in the early detection of glaucoma and reduce the incidence of consequent adverse outcomes.

## Methods

### Study participants

This study used a purposive sampling method, and the sample size was based on the principle of information saturation. Participants were selected from inpatients admitted to the glaucoma ward of a tertiary eye hospital in Tianjin, China, from January 2021 to April 2021. The inclusion criteria were as follows: intraocular pressure >21 mmHg; glaucomatous optic nerve changes and glaucomatous visual field defects in one or both eyes at the first ophthalmological visit; requirement for glaucoma treatment; and ability and willingness to express the full details of the process leading to the delay of their first ophthalmological consultation.

### Research method

#### Theoretical basis

**Figure 1:**
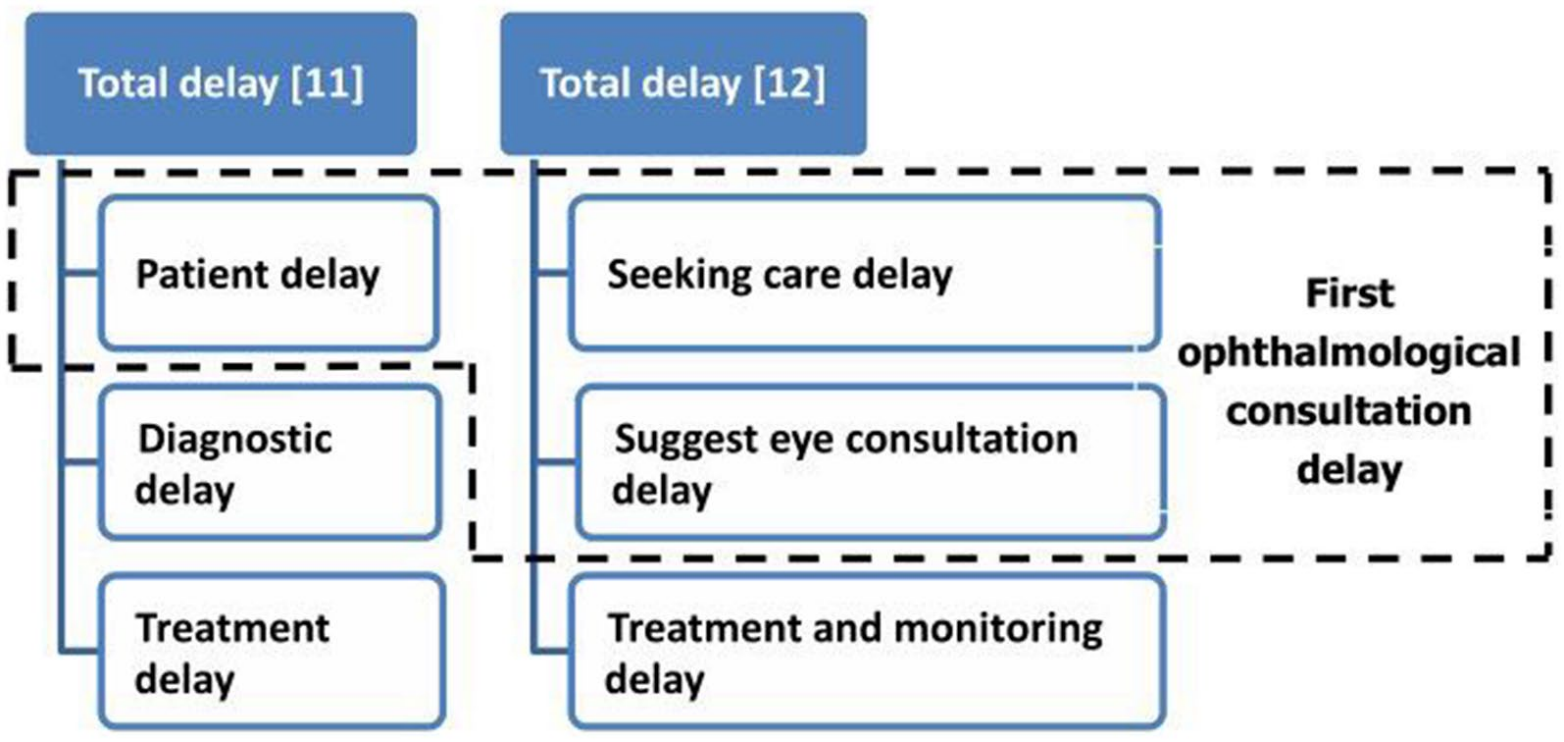
Theoretical basis of first ophthalmological consultation delay. The theoretical basis for our investigation was that the first ophthalmological consultation delay can be caused by both patient delay and seeking care delay.

The theoretical basis for our investigation was that the first ophthalmological consultation delay can be caused by both patient delay and seeking care delay; this was supported by the results of our literature review (11, 12) (Figure 1). Thus, this theoretical basis was used to ensure that the interview content reached saturation.

#### Interview outline

Semi-structured interviews were used to collect data. A preliminary interview outline was formulated based on our study objective and literature review. We then consulted one qualitative research expert, two nursing master graduate students, two glaucoma experts, and one glaucoma ward nurse to revise the interview outline. Two glaucoma patients who met the inclusion criteria of this study were selected via a convenience sampling method for a pre-interview, which was performed to ensure that the interview questions were clear and understandable. The interview outline was subsequently finalized.

The interview questions were as follows: (1) “Do you know the disease from which you suffer? What is it?”; (2) “What troubles and difficulties did you encounter in the process of detecting symptoms, determining that you were sick, deciding to seek care, choosing a hospital, and starting treatment and monitoring?”; (3) “Please recall the influence of your work, economic state, and family life on your first ophthalmological consultation and medical treatment”; and (4) “Please recall the influence of your knowledge level and medical treatment attitude on first seeking ophthalmological consultation.”

#### Data collection method

The interview was conducted in the glaucoma ward. Before starting the interview, the purpose of the study was explained to the patients. It was clarified that the interview recording was to be used only for medical research and that the research results would be summarized anonymously. After obtaining consent, the interview process, including non-linguistic expressions such as expression, tone, and gesture, were recorded. The interview process was based on the principle that patients fully expressed the details of their experience with delaying their first ophthalmological consultation. The interview was ended when no new information was procured.

#### Data analysis method

After the interview, Colaizzi’s seven-step method (15) was used to analyze the data. Two researchers listened to the recordings after each interview was completed, to determine the degree of saturation of the data. After confirming data saturation, transcription personnel transcribed the recordings for consistency calibration. The text was carefully and repeatedly read and subsequently imported into Nvivo12^1^ to identify significant statements, formulate meaning, cluster themes, develop exhaustive descriptions, and produce the fundamental structure.

## Results

Forty-six patients with glaucoma, who delayed their first ophthalmological consultation and subsequently received glaucoma treatment, were enrolled in this study. There were 29 primary angle closure glaucoma (PACG) patients (11 males, 18 females) with a mean age of 64.07 ± 7.33. The delay time ranged from 3 to 24 months with a median of 7 (3, 14) months, and most of the caregivers were spouses (21/29, 72.41%). Among these 29 PACG patients, 10 were retired, 8 were unemployed and had pensions, and 11 were other professionals, covering multiple occupations. In terms of medical insurance, 27 patients had medical insurance and only 2 patients did not. The degree of visual field impairment from mild to severe was as follows: 5 patients with binocular paracentral scotoma (PS), 4 patients with monocular PS and the other eye nasal step (NS), 8 patients with monocular PS and the other eye wedge-shaped depression (WSD), and 3 patients with monocular NS and the other eye WSD. There were 17 primary open angle glaucoma (POAG) patients (9 males, 8 females) with a mean age of 67.29 ± 7.30, and there was no statistical difference in age between PACG patients and POAG patients (independent sample t test, *p* = 0.156). The delay time ranged from 3 to 24 months with a median of 15 (10, 20.5) months. There was a significant difference between the two groups in delayed first ophthalmological consultation (Wilcoxon rank sum test, *p* = 0.008). Most of the caregivers were spouses (13/17, 76.47%), which is similar to that of PACG patients. Among these 17 POAG patients, 7 were retired, and 10 were other professionals, covering multiple occupations, but there were no unemployed individuals. The degree of visual field impairment from mild to severe was as follows: 2 individuals with monocular PS and NS in the other eye, 2 individuals with binocular NS, 2 individuals with monocular NS and WSD in the other eye, and the other 11 individuals with moderate to severe visual field injury (11/17, 64.71%). The general information of the respondents is shown in Table 1.

**Table 1:** Baseline patient data (*n* = 46).

NO.	Type of glaucoma	Sex	Age (years)	First ophthalmological consultation delay (months)	Affected eye and visual field	Caregiver	Profession	Medicare (yes/no)
N1	POAG	M	66	19	OD NS; OS WSD	Spouse	Government official	Yes
N2	POAG	M	67	7	OD NS; OS PS	Spouse	Teacher	Yes
N3	PACG	F	51	24	OD PS; OS TVF	Spouse	Blue-collar laborer	Yes
N4	PACG	F	73	10	OD WSD; OS NS	Spouse	Retiree	Yes
N5	POAG	M	65	8	OD NS; OS PS	Spouse	Farmer	No
N6	PACG	F	74	24	OD WSD; OS PS	Spouse	Unemployed	Yes
N7	POAG	M	73	24	OD TVF; OS WSD	Son	Retiree	Yes
N8	PACG	M	55	3	OD PS; OS PS	Spouse	Kitchen worker	Yes
N9	PACG	M	61	23	OD WSD; OS TVF	No one	Retiree	Yes
N10	POAG	F	67	13	OD WSD; OS WSD	Spouse	Farmer	Yes
N11	POAG	F	77	22	OD WSD; OS TVF	Spouse	Retiree	Yes
N12	PACG	F	65	10	OD NS; OS WSD	Spouse	Unemployed	Yes
N13	PACG	M	68	3	OD NS; OS PS	No one	Farmer	Yes
N14	POAG	M	73	24	OD TVF; OS NS	Spouse	Policeman	Yes
N15	PACG	F	54	3	OD PS; OS PS	Spouse	Accountant	Yes
N16	PACG	F	55	3	OD PS; OS PS	Spouse	Tailor	No
N17	PACG	M	73	20	OD WSD; OS WSD	Son	Retiree	Yes
N18	POAG	M	53	10	OD NS; OS NS	Spouse	Attendant	Yes
N19	PACG	F	68	21	OD TVF; OS TVF	Daughter	Retiree	Yes
N20	PACG	F	64	18	OD WSD; OS WSD	Spouse	Farmer	Yes
N21	POAG	M	76	14	OD NS; OS TVF	Son	Retiree	Yes
N22	POAG	F	72	19	OD TVF; OS WSD	Son	Retiree	Yes
N23	PACG	M	70	3	OD PS; OS WSD	Son	Retiree	Yes
N24	PACG	F	59	6	OD WSD; OS PS	Spouse	Unemployed	No
N25	PACG	F	57	17	OD PS; OS WSD	Spouse	Barber	Yes
N26	PACG	F	70	5	OD WSD; OS PS	Spouse	Unemployed	Yes
N27	POAG	M	52	3	OD NS; OS NS	Spouse	Mechanic	Yes
N28	PACG	M	59	5	OD PS; OS NS	Spouse	Policeman	Yes
N29	PACG	F	73	11	OD TI; OS NS	Spouse	Unemployed	Yes
N30	PACG	F	78	9	OD TI; OS TVF	Spouse	Retiree	Yes
N31	POAG	F	67	15	OD WSD; OS WSD	Spouse	Retiree	Yes
N32	PACG	F	60	5	OD WSD; OS NS	No one	Retiree	Yes
N33	PACG	M	63	3	OD NS; OS PS	No one	Unemployed	Yes
N34	POAG	M	61	10	OD NS; OS TVF	Spouse	Professor	Yes
N35	POAG	F	62	17	OD TVF; OS NS	Spouse	Farmer	No
N36	PACG	M	70	3	OD PS; OS NS	Spouse	Retiree	Yes
N37	POAG	F	72	16	OD WSD; OS WSD	Spouse	Retiree	Yes
N38	PACG	F	64	3	OD PS; OS PS	Spouse	Unemployed	Yes
N39	PACG	F	67	8	OD WSD; OS PS	Spouse	Unemployed	Yes
N40	PACG	M	61	6	OD PS; OS PS	Spouse	Blue-collar laborer	Yes
N41	PACG	F	63	7	OD WSD; OS NS	Spouse	Retiree	Yes
N42	PACG	M	62	5	OD TVF; OS WSD	No one	Engineer	Yes
N43	PACG	F	50	10	OD PS; OS WSD	Spouse	Journalist	Yes
N44	POAG	F	75	12	OD WSD; OS NS	Son	Retiree	Yes
N45	PACG	M	71	8	OD PS; OS WSD	Spouse	Retiree	Yes
N46	POAG	F	66	23	OD IT; OS WSD	Spouse	Farmer	Yes

M, male; F, female; POAG, primary open-angle glaucoma; PACG, primary angle-closure glaucoma; OD, Right eye; OS, Left eye, PS, paracentral scotoma; NS, nasal step; WSD, wedge-shaped depression; TVF, tube visual field; TI, temporal island

Analysis of the interview data resulted in the extraction of 1,396 effective semantic reference points, duplicate semantic reference points were 269 items. Four major themes and sixteen sub-themes were formed. The four themes were related to aspects of disease recognition, symptom confusion, hazard cognition, and the support system, and were defined as follows: (1) occult symptoms are difficult to identify; (2) insufficient knowledge and understanding of glaucoma-related risks and harm; (3) perceived difficulties in accessing medical care; and (4) inadequate support system. The specific interview content is shown in Table 2.

**Table 2:** Major themes: factors involved in the delay to first ophthalmological consultation by patients with primary glaucoma.

Major themes	Content of the interview
Symptoms are occult and difficult to identify	“In the beginning, I always had migraine, a burst of pain; I thought that was nerve pain!” (N15)
	“When I worked in a restaurant, I often felt dizzy and struggled to see things. The room was so hot that I thought I was *Shanghuo*.” (N18)
	“I did not know I had glaucoma. I visited a doctor, every time the medical eye examination was normal.” (N34)
Insufficient knowledge and understanding of glaucoma-related risk and harm	“Many years ago my eyes pained; I had no pain over the past 2 years. Recently I had eye pain for 2 days, but I did not mind.” (N6)
	“I do not know the purpose of checking the visual field; my visual acuity was not affected, and I thought my eyes were normal. But visual field damage is so serious” (N22)
Perceived difficulties in accessing medical care	“I do not have pension or healthcare insurance. I rely on my husband’s pension of 3,000 Chinese Yuan per month; how can I go to see a doctor with this amount of money?” (N4)
	“My home is far from the hospital, more than 30 miles. The only way to get there is to take a shuttle car, and then take a bus.” (N5)
Inadequate support system	“My eldest daughter is a teacher and her work keeps her very busy; her children are studying for their college entrance examination and have no time to take me to see the doctor.” (N13)
	“We are not registered and rarely go to the hospital. We do not know how to register online and have to wait for our child to do this for us.” (N45)

### Glaucoma symptoms are occult and difficult to identify

The first major theme included five sub-themes, which covered the patient ignoring the prodrome and common pitfalls. Factors involved in ignoring the prodrome included atypical symptoms, low medical care level, and a lack of health education. Common pitfalls included thinking that symptoms were due to systemic disease or the natural aging process. These five sub-theme items and interview records are shown in Table 3.

**Table 3:** Major theme 1: glaucoma symptoms are occult and difficult to identify.

Sub-themes	Content of the interview
Symptoms are not typical	“I went to the toilet in the middle of the night. Wow! My eyes blurred; I thought that I got up too fast. I did not care, and went back to sleep, got up in the morning, and it was all right.” (N16)
	“Blurred vision was not severe and did not affect my work, so I did not buy any medicine. I had been deceived that it was *Shanghuo*, and did not pay much attention.” (N27)
	“I was informed of glaucoma in the hospital. I went to the cataract department for surgery, and the doctor told me that I should make an appointment with the glaucoma specialist. Only then did I know that I had glaucoma.” (N14)
Medical care level is poor	“The doctor of my county hospital checked my eyes, and she said my eyes were too dirty, and that I needed to massage my eyes. I immediately agreed! But the massage was very uncomfortable, was so uncomfortable that I burst into tears!” (N33)
	“The doctor of your hospital was very sure and said that I had glaucoma. I lived in Tianshui, Gansu province, where there was no local ophthalmologist. Doctor Ji of your hospital came to Tianshui once every month.” (N1)
Lack of health education	“I never surf the internet. I do not understand the words. My medical knowledge is very poor.” (N20)
	“I do not know anything about glaucoma. Take cataract for example, now everyone knows about that, but people really lack glaucoma knowledge.” (N28)
Symptoms are affected by systemic disease	“I told my mother-in-law that I could not see anything. She said it was a side-effect of chemotherapy” (N3)
	“It’s like catching a cold. I mistakenly thought I had a cold, and my blood pressure was so high that I could not see.” (N11)
	“I’m old and I have a headache. I suspected that I had a tumor in my head. I got an MRI, but there was nothing wrong.” (N5)
Symptoms are mistaken for symptoms of natural aging	“I have blurred vision. Since I was young, I have been weaving gloves, pasting matchboxes, and making toothbrushes. I thought my eyes were too tired because I used them too much.” (N37)
	“When I was young, I had strong immunity and I was not easy to get sick. Now, I am old, and my immunity is weakened. That was why my eyes were bad.” (N9)

### Insufficient knowledge and understanding of glaucoma-related risk and harm

The second major theme included three sub-themes, which covered the following: a lack of awareness of the symptoms and harm caused by the disease, as well as neglected factors, such as self-medication. Common cognitive deficiencies included not being aware of the harm caused by glaucoma, poor willingness to seek medical care, and self-medication habits. The content of the three sub-themes and interview records are shown in Table 4.

**Table 4:** Major theme 2: insufficient knowledge and understanding of glaucoma-related risk and harm.

Sub-themes	Content of the interview
Not aware of glaucomatous harm	“I do not understand, I did not think it was so severe; anyway, I feel the eyes are covered but not serious.” (N2)
	“When I was a child, I had good vision. I was fine all my life. I did not know glaucoma was so serious.” (N27)
Poor willingness to seek medical care	“I also love to inquire. A neighbor was performed glaucoma surgery, I think the effect was not good. He said the treatment had no curative effect. I believed him, so I always delayed seeing a doctor.” (N19)
	“I grew up in the countryside. When I got sick, I had no medicine. I had not gone to the hospital for decades.” (N26)
Self-medication habits	“I used the eye drops that my children had brought back from Japan! They were meant for treatment of visual fatigue, and I felt a little better!” (N8)
	“My husband said I had cataracts, so I went to the pharmacy to buy eye drops. I used the eye drops for 2 years.” (N46)

### Perceived difficulties in obtaining medical care

The third major theme included five sub-themes, which covered marked economic pressure, a lack of trust in seeking medical treatment, long travel distance required to access medical care, a lack of glaucoma specialists or examination facilities, and limited medical resources. The contents of the five sub-themes and interview records are shown in Table 5.

**Table 5:** Major theme 3: perceived difficulties in obtaining medical care.

Sub-themes	Content of the interview
Marked economic pressure	“I was born in a village. When I married my husband, I came to live in the city. I had no retirement fee, and no money to see a doctor!” (N29)
	“Medical insurance does not cover the outpatient expenses. It usually takes me more than 10,000 Chinese Yuan a year to pay for my systemic disease (diabetes, hypertension, heart disease). So, I did not check for glaucoma before.” (N39)
Lack of trust in seeking medical treatment	“I do not really believe what the doctor said, I am afraid that the doctor wants to prescribe more medicine and do more surgery. And your hospital has interns, so I am even more afraid that the doctor will be selfish.” (N7)
	“My father is also a doctor, not of ophthalmology, and he advocated going to other hospitals and get comprehensive opinions from different hospitals.” (N43)
Medical institutions is far away	“It was a long way away. It took more than an hour to drive here. There was no vacant parking space in the hospital, my son stopped his car two kilometers away, walked to the hospital and rented a wheelchair. Then, he walked back and picked up me with a wheelchair from parking to hospital. It took me another hour.” (N17)
	“It took me 23 h to come here by train, it will not save much time even by plane (sigh).” (N42)
No glaucoma specialist or examination facilities	“Work unit physical examination and community physical examination do not involve the eyes, the county hospital only checks the common diseases.” (N24)
	“Doctors in the village only screen cataracts; they can do cataract surgery, but not glaucoma surgery.” (N35)
Limited medical resources	“I made an appointment with my smartphone. For next week’s clinic, the fastest you can see a doctor is in 3 or 4 days.” (N25)

### Inadequate support system

The fourth major theme included three sub-themes, which covered the following: being unable to see the doctor by themselves, being too busy with work or life; having an inadequate family support system. The three sub-theme contents and interview records are shown in Table 6.

**Table 6:** Major theme 4: inadequate support system.

Sub-themes	Content of the interview
Inability to see the doctor by themselves	“I need my son when I go to see a doctor, not because of my poor eyesight: I cannot make an appointment online and pay electronically, I cannot do it.” (N31)
	“I cannot go to the hospital by myself. I do not know where to go. My daughter, daughter-in-law, son-in-law and my son, they take turns in going to hospital with me.” (N12)
Being busy with work or life	“I just got a job 6 months ago. Every colleague has his own task, and there is nobody to spare. I got the position by other’s recommendation. I am too embarrassed to ask for leave, ah!” (N40)
	“I pick up my grandson from school every day. I have to cook for my family and have no time to see a doctor.” (N44)
Inadequate family support system	“Since last year, I felt something like hair floating in front of my eyes, but I did not have long hair … I told my husband, and he did not care.” (N10)
	“My eyes were uncomfortable for 2 weeks, and my children did not have time at that time. Now, they are free and they took me to the hospital.” (N23)

**Figure 2:**
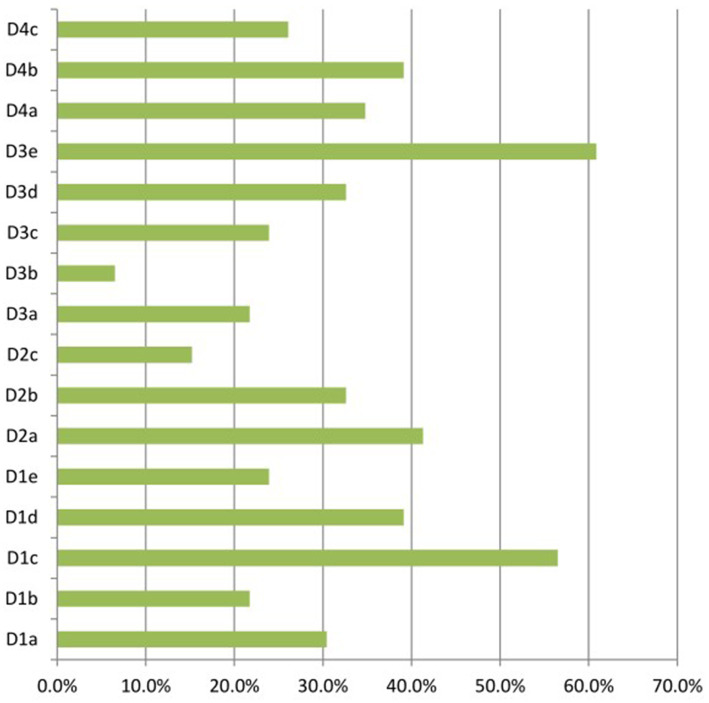
The first ophthalmological consultation delay factors. D1: symptoms are occult and difficult to identify (D1a: symptoms are not typical; D1b: medical care level is poor; D1c: lack of health education; D1d: symptoms are affected by systemic disease; D1e: symptoms are mistaken for symptoms of natural aging); D2: insufficient knowledge and understanding of glaucoma-related risks and harm (D2a: not aware of glaucomatous harm; D2b: poor willingness to seek medical care; D2c: self-medication habits); D3: perceived difficulties in accessing medical care (D3a: marked economic pressure; D3b: lack of trust in seeking medical treatment. D3c: medical institutions is far away; D3d: no glaucoma specialist or examination facilities; D3e: limited medical resources); D4: inadequate support system (D4a: inability to see the doctor by themselves; D4b: being busy with work or life; D4c: inadequate family support system).

All the above first ophthalmological consultation delay factors are shown in Figure 2.

## Discussion

In this study, we interviewed 46 patients with primary glaucoma who had delayed seeking their first ophthalmological consultation. From the general data of the patients, the visual field damage in PACG was milder, with 20 patients experienced mild to moderate visual field damage (20/29, 68.97%), while only 6 POAG patients (6/17, 35.29%) had mild to moderate visual field damage. This may be due to the longer delay and the more insidious symptoms in POAG. In this study, the median of delayed medical visit time in POAG patients was significantly greater than that of PACG patients, indicating that POAG patients had longer delay in first ophthalmological consultation. Therefore, it is necessary to conduct more in-depth research on the delayed treatment of POAG patients in the future. Ophthalmologists should take more time to consider how to reduce the delay in POAG. Although PACG had a slightly shorter delay in seeking ophthalmological consultation and less severe visual field damage in this study, a targeted intervention in PACG patients with mild to moderate visual field damage would better maintain their residual visual function and effectively avoid blindness. Therefore, more attention should be paid to delayed seeking of medical treatment in both types of glaucoma. In addition, although no jobless people were found in POAG group, the POAG patients presented greater consultation delay, indicating economic level may not be the determinant factor of delayed medical attendance.

From the results of the 46 patient interviews, the most important reasons for the delay was the difficulty in identifying glaucoma symptoms, which was recounted by 31 patients (31/46, 67.39%). Other common reasons for the delay in seeking medical treatment in ophthalmology departments included the lack of awareness of the harm caused by glaucoma, perceived difficulties in obtaining medical treatment, and an inadequate support system. In contrast to previous studies, we used an in-depth interview process to address disease cognition, life rhythm, work intensity, medical habits, local medical resources, physical care, economic status, family support, and other aspects. Thus, our results provide a more accurate reflection of the primary causes of first ophthalmological consultation delay.

### Improve disease awareness of both doctors and patients through social media and standardized training

Glaucoma damages the optic nerve through high intraocular pressure, which subsequently leads to an irreversible visual field defect and eventual blindness (1). In the process of visual field damage, glaucoma commonly causes symptoms such as nose pain, headache, and a transient decline in visual acuity. Most patients are unaware that they have glaucoma in the early stages of the disease. Indeed, our results indicated that insufficient knowledge and understanding of glaucoma-related harm was a major issue, consistent with the findings of previous studies (16). Due to the atypical symptoms, 21 patients (21/46, 45.65%) did not realize the importance and urgency of ophthalmological consultation. Thus, there is an immediate need to popularize the knowledge of glaucoma. Previous studies (17, 18) have called for the problem of glaucoma perception to be addressed through the mass media. In addition, symptom identification and the early diagnosis of glaucoma are not focal points in current standardized training programs for non-ophthalmic medical staff. Komolafe et al. (19) recommended the strengthening of glaucoma knowledge among medical staff, in order to educate the general population about glaucoma and the importance of avoiding ophthalmological consultation delay. In the present study, 14 patients (11 POAG and 3 PACG) complained of undetected glaucoma-related symptoms, highlighting the urgent need to popularize knowledge about glaucoma, especially for POAG patients. Celebi (20) recommended the strengthening of community education. A feasible public health strategy would be to screen high-risk groups, such as older individuals and those with a family history of glaucoma in the community, on the premise of enhancing the awareness of glaucoma.

### Use internet resources to carry out glaucoma health education projects and change medication habits

While the initial symptoms of glaucoma are atypical, the visual field damage is progressive, and causes a sharp decline in patients’ quality of life and increases treatment costs, thus emphasizing the value of early intervention (21, 22). Progressive glaucoma is associated with decreased reading ability and mobility, inability to drive and work (23–25), as well as adverse psychological effects (26, 27). In this study, 19 patients (19/46, 41.30%) had delayed their first ophthalmological consultation for 12 months or longer; this included 12 cases of primary open-angle glaucoma and 7 cases of primary angle-closure glaucoma. While the symptoms of the two types of glaucoma differ, a general lack of awareness of disease harm typically leads to a delay in seeking medical treatment, regardless of glaucoma type. Indeed, consultation delay remains a major problem and challenge for the medical industry.

The first step in overcoming this challenge would be to increase the willingness of high-risk groups with glaucoma to actively seek medical care (28). In the early stage of glaucoma, it is difficult for most patients to comprehend the potential serious decline in quality of life that would be caused by visual field damage (29). In addition, the attitude toward the disease needs to be changed through health education (30). Twenty-six patients (26/46, 56.52%) mentioned “lack of health education” in the interviews, urging us to promote the quality of patients’ health education. At present, internet medical care services are available in various forms (31–33), particularly in low- and middle-income countries and regions. We suggest that special glaucoma health education internet projects should be increased, health education for high-risk groups should be strengthened, and habits of avoiding medical care consultation should be changed.

### Medical union and medical multi-point practice for resolving practical difficulties

The medical union model of some developing countries requires that all tertiary hospitals participate in and play a leading role in regional medical care; furthermore, health services should be responsible for medical management of the region (34). The imbalance of medical resources, long waiting time for medical resources, long travel distances to medical treatment facilities, and low rate of medical insurance reimbursement should be resolved. Medical unions may be particularly effective in resolving issues related to the marked economic pressures and long travel distances faced by patients, as well as limited medical resources. Indeed, 28 patients (28/46, 60.87%) in the present study cited insufficient medical resources as the cause of their delay in obtaining ophthalmology treatment. A previous study reported that the medical union model was able to remedy registration difficulties and long waiting times for ophthalmological consultation (35).

However, in the process of promoting the work of the medical union, issues with insufficient medical staff and an inability to achieve homogenization will exacerbate the lack of trust in medical treatment (36). In the process of promoting the multi-site practice of medical staff, some countries have also set up prescribing rights for specialized nurses in community hospitals, which to some extent solves problems such as insufficient manpower, insufficient medical resources, and the fact that many older people cannot use the online appointment registration system (37, 38). The promotion of medical technology from tertiary hospitals to primary hospitals is conducive to glaucoma examination, ensures homogenization, and increases the trust of patients through the provision of high-quality medical services.

### Improving the whole social medical support system through multiple channels

Three themes in the present study highlighted an inadequate support system: the inability of the patient to see a doctor by themselves; being too busy with work or life; and having an inadequate family support system. To solve these three problems, it is necessary to establish a multi-channel social-family support model. Many countries currently implement the checkout mode of seeking medical treatment before payment, which can effectively resolve the issue of patients who are unable to see a doctor alone. Although the “medical and postpayment” model and electronic payment channels in various countries have achieved some successful results, many problems remain (39, 40). Improvements are still required in terms of the strength and integrity of the doctor-patient relationship, the number of medical and auxiliary personnel, and communication to patients at key points, such as inspection item notification and cost settlement. At the same time, social health science should be popularized to enhance the awareness of glaucoma and to enhance family support. As middle-aged patients with glaucoma are particularly affected by a busy work schedule, fast pace-of-life, and other problems, internet hospitals should be promoted (41, 42). Internet hospitals would allow patients who are proficient in using the internet to quickly and conveniently complete preliminary consultation and screening for glaucoma, thereby reducing the incidence of ophthalmological consultation delay.

### Limitations

We acknowledge the limitations of this study. First, this was a preliminary pilot study and the sample size was small. Second, the research participants in this study were older individuals with primary glaucoma. As older patients may be less able to perceive symptoms and typically experience more practical difficulties, they are also more prone to ophthalmological consultation delay than younger patients. In addition, the incidence of primary glaucoma is higher among older individuals. A study with larger sample size (especially younger patients with glaucoma) is needed to address the cause of consultation delay among young patients.

## Conclusion

A delay in ophthalmological consultation and treatment for glaucoma leads to an irreversible damage to visual function. In this study, we elucidated real-life reasons for the delay of ophthalmological consultation. This information can be used as the basis for the continuous improvement of management protocols and systems to prevent glaucoma-related blindness.

## Data availability statement

The original contributions presented in the study are included in the article/supplementary material, further inquiries can be directed to the corresponding author.

## Ethics statement

This study was reviewed and approved by the Medical Ethics Committee of Tianjin Medical University Eye Hospital (2020KY(L)-48). All participants provided written informed consent.

## Author contributions

HL interviewed patients with glaucoma and was a major contributor in writing the manuscript. CC, ZC, QiL, and QuL transcribed the recording into the text. Data analysis was performed by HL and WL. All authors contributed to the article and approved the submitted version.

## Funding

This study was funded by the Tianjin Key Medical Discipline (Specialty) Construction Project (TJYXZDXK-037A). The funder has no roles in the design of the study and collection, analysis, and interpretation of data and in writing the manuscript.

## Conflict of interest

The authors declare that the research was conducted in the absence of any commercial or financial relationships that could be construed as a potential conflict of interest.

## Publisher’s note

All claims expressed in this article are solely those of the authors and do not necessarily represent those of their affiliated organizations, or those of the publisher, the editors and the reviewers. Any product that may be evaluated in this article, or claim that may be made by its manufacturer, is not guaranteed or endorsed by the publisher.

## Footnotes

^1^ https://www.qsrinternational.com/nvivo-qualitative-data-analysis-software/home
